# Development of Antibody and Antibody Fragment-Based Radiopharmaceuticals for Cancer Imaging and Therapy

**DOI:** 10.1007/s13139-025-00969-3

**Published:** 2025-12-02

**Authors:** Chi Soo Kang

**Affiliations:** Division of Applied RI, Korea Institute of Radiological and Medical Sciences, Seoul, 01812 Republic of Korea

**Keywords:** Antibody, Based radiopharmaceuticals, Cu, Cu-64, Zr-89, I-124, Lu-177, Ac-225, Bi-213, Pb-212

## Abstract

Cancer has been the leading cause of death and imposing tremendous socioeconomical pressure worldwide. It is considered the most difficult disease to tackle and major obstacle for extending life expectancy. Radiopharmaceuticals in the field of cancer diagnosis and therapy have drawn a lot of research interest past years due to clinical success, e.g. [^177^Lu]Lu-PSMA-617 (Pluvicto®) for the treatment of metastatic castration-resistant prostate cancer (mCRPC). Antibody-based radiopharmaceuticals are the most studied in the field of nuclear medicine for cancer attributed to antibody’s great tumor targeting ability. In this manuscript, recent preclinical studies of developing imaging and therapeutic radiopharmaceuticals based on antibody or antibody fragments, including affibody and nanobody, are introduced.

## Introduction

Cancer is the leading cause of death among noncontagious diseases and producing millions of deaths each year worldwide and imposing a heavy socioeconomical burden on each country. Traditional cancer treatment options include surgical removal, radiation therapy, and chemotherapy. However, they are either limited to localized solid tumors or causing side effects by damaging normal cells and tissues leading to reduced patient’s quality of life. Vast number of cancer-targeted therapeutic agents including small molecule drugs, therapeutic antibodies, and antibody–drug-conjugates (ADCs) has been developed and showed clinical success. However, a large fraction of patients, who administered with those therapeutics, develops drug resistance and cancer becomes refractory necessitating more effective therapeutic methods. Targeted radionuclide therapy (TRT) has drawn a lot of attention past years due to the clinical success and marketization of [^177^Lu]Lu-PSMA-617 (Pluvicto®) for the treatment of metastatic castration-resistant prostate cancer (mCRPC), and since then various tumor-specific biomarkers and targeting moieties have been investigated for TRT. Radiopharmaceuticals for diagnosis of cancer also have been extensively investigated because patient selection is extremely important for efficient treatment leading to better prognosis. Numerous radiopharmaceuticals for diagnostic and therapeutic purposes using different tumor-targeting moieties, and among the delivery molecules, antibody and antibody fragments have been the most utilized biomolecules, along with peptide, due to its high binding affinity. Table 1 provides examples of radiolabeled antibodies and antibody fragments that have been in clinical studies. In this review, recent studies in the development of diagnostic and therapeutic radiopharmaceuticals using antibody and antibody fragments are introduced. In the first part, studies about developing imaging radiopharmaceuticals labeled with positron-emitting radionuclides such as Cu-64, Zr-89, and I-124 are presented, and the second part deals with researches for the development of therapeutic radiopharmaceuticals utilizing Lu-177, Ac-225, Bi-213 and Pb-212.

**Table 1:** Radiolabeled antibodies and antibody fragments in clinical studies for diagnostic and therapeutic purposes

Function	Probe	Target	Targeting moiety	Cancer types	Ref
Diagnostics	[^64^Cu]Cu-DOTA-Trastuzumab	HER2	mAb	Metastatic breast cancer	[1]
	[^89^Zr]Zr-Pertuzumab	HER2	mAb	Breast cancer	[2]
	[^124^I]I-Trastuzumab	HER2	mAb	Gastric cancer	[3]
	[^68^ Ga]Ga-HER2-Nanobody	HER2	Nanobody	Breast cancer	[4]
	[^64^Cu]Cu-DOTA-Patritumab	HER3	mAb	Colonic, endometrial, pancreatic, esophageal, non-small lung cancers	[5]
	[^89^Zr]Zr-Lumretuzumab	HER3	mAb	Rectal, colon, head and neck, ovarian, esophageal, breast cancer	[6]
	[^89^Zr]Zr-DFO-Nimotuzumab	EGFR	mAb	U87MG	[7]
	[^89^Zr]Zr-Panimotuzumab	EGFR	mAb	Ameloblastoma	[8]
	[^89^Zr]Zr-Cetuximab	EGFR	mAb	Head and neck and lung cancer	[9]
	[^89^Zr]Zr-Atezolizumab	PD-L1	mAb	Bladder cancer, non-small cell lung cancer, triple-negative breast cancer	[10]
	[^89^Zr]Zr-Envafolimab	PD-L1	Nanobody	Lung, pancreatic, colorectal cancer, melanoma, neuroendocrine tumor	[11]
	[^89^Zr]Zr-Pembrolizumab	PD-1	mAb	Melanoma, non-small cell lung cancer	[12]
	[^89^Zr]Zr-Crefmirlimab	CD8	Minbody	Melanoma, hepatocellular carcinoma	[13]
	[^89^Zr]Zr-Girentuximab	CA9	mAb	Clear-cell renal cell carcinoma	[14]
	[^89^Zr]Zr-huJ591	PSMA	mAb	Prostate cancer	[15]
	[^89^Zr]Zr-Df-IAB2M	PSMA	Minibody	Prostate cancer	[16]
Therapeutics	[^177^Lu]Lu-Trastuzumab	HER2	mAb	Breast cancer	[17]
	[^212^Pb]Pb-Trastuzumab	HER2	mAb	Peritoneal metastases	[18]
	[^90^Y]Y-Epratuzumab	CD22	mAb	Lymphoblastic leukaemia	[19]
	[^90^Y]Y-Ibritumomab	CD20	mAb	Non-Hodgkin Lymphoma	[20]
	[^225^Ac]Ac-Lintuzumab	CD33	mAb	Acute myeloid leukemia	[21]
	[^177^Lu]Lu-Lilotomab	CD37	mAb	Non-Hodgkin lymphoma	[22]
	[^186^Re]Re-Bivatuzumab	CD44	mAb	Head and neck squamous cell carcinoma	[23]
	[^177^Lu]Lu-Girentuximab	CA9	mAb	Renal cell carcinoma	[24]
	[^131^I]I-3F8	GD2	mAb	Brain and leptomeningeal cancer	[25]
	[^177^Lu]Lu-J591	PSMA	mAb	Prostate cancer	[26]
	[^131^I]I-Sibrotuzumab	FAP	mAb	Colorectal, non-small cell lung cancer	[27]
	[^131^I]I-Labetuzumab	Carcinoembryonic antigen	mAb	Colorectal cancer	[28]

Abbreviations: *mAb* Monoclonal Antibody; *HER2* Human Epidermal Growth Factor Receptor 2; *EGFR* Epidermal Growth Factor Receptor; *CA9* Carbonic Anhydrase 9; *PD-L1* Programmed Death-Ligand 1; *PD-1* Programmed Cell Death Protein 1; *CD8* Cluster of Differentiation 8; *PSMA* Prostate Specific Membrane Antigen; *FAP* Fibroblast Activation Protein; *CD22* Cluster of Differentiation 22; *CD33* Cluster of Differentiation 33; *CD37* Cluster of Differentiation 37; *CD44* Cluster of Differentiation 44; *GD2* Disialoganglioside

## Antibody-Based Radiopharmaceuticals for Cancer Diagnosis

Intact IgG type antibody has been extensively utilized in nuclear imaging of cancer. Conventional IgG is composed of two identical heavy and light chains, where the heavy chain consists of three constant domains (C_H_1, C_H_2, C_H_3) and a variable domain (VH) and the light chain consists of one constant domain (CL) and one variable domain (VL). Due to its size (~ 150 kDa), it is difficult for intact IgG to be excreted via glomerular filtration (clearance cut-off is around 60 kDa) [29]. Moreover, interaction between Fc domain of heavy chain with Fc receptor in neonatal epithelial cells further hinders fast excretion resulting in low tumor to background ratio [29]. Consequently, radionuclides with long half-life such as Zr-89 (T_½_ = 3.2 days) or I-124 (T_½_ = 4.2 days) should be the choice for developing IgG-based diagnostic radiopharmaceutical [30]. To address the limitations of IgG as a delivery platform, various engineered antibody fragments have been developed. Minibody is a dimer of single-chain variable-fragment (scFv) fused with heavy chain C_H_3 (scFv-C_H_3) having molecular weight around 80 kDa, and diabody is a divalent version of the scFv without C_H_3 domain having molecular weight approximately 60 kDa. They have relatively faster pharmacokinetics compared to intact IgG requiring radionuclides with intermediate half-life, for example, Cu-64 (T_½_ = 12.7 h) or Y-86 (T_½_ = 14.7 h) [30]. A scFv is composed of variable light and variable heavy domains connected by a peptide bond having molecular weight around 25 kDa, and nanobody is the variable domain of heavy chain of a camelid heavy-chain-only antibodies (HCAbs) with molecular weight of approximately 15 kDa. They exhibit extremely fast pharmacokinetics, and imaging radionuclides with short half-life such as Ga-68 (T_½_ = 68 min) or F-18 (T_½_ = 110 min) are normally utilized for the development of scFv- or nanobody-based radiopharmaceuticals [30].

### Cu-64

Cu-64 has been extensively studied as an imaging radionuclide for positron emission tomography (PET) due to its favorable physical characteristics. Relatively longer half-life (12.7 h) compared to other commonly used imaging radionuclides, such as F-18 (T_½_ = 110 min) and Ga-68 (T_½_ = 68 min), allows extended tumor imaging, and low positron energy (E_mean_ = 278 keV) results in short positron range leading to better image resolution [31]. Moreover, well-established coordination chemistry of Cu(II) has led to the development of various bifunctional chelators providing high thermodynamic stability and kinetically inert Cu-64-labeled complexes [32]. There have been numerous researches exploiting Cu-64-labeled antibodies or antibody fragments as a tumor imaging radiopharmaceuticals.

**Fig. 1:**
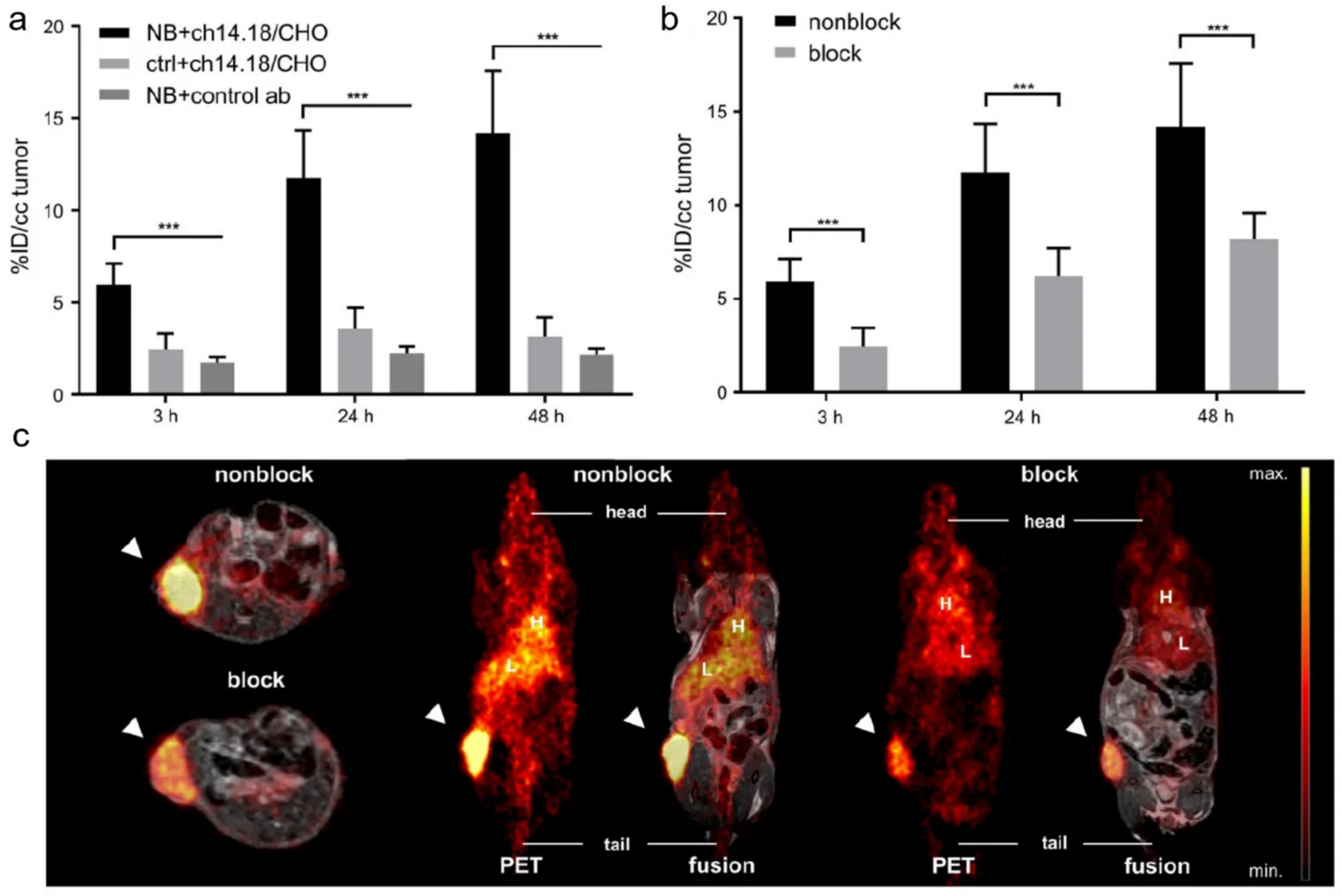
In vivo accumulation of [^64^Cu]Cu-DOTAGA-ch14.18/CHO. (**a**) %ID/cc tumor of [^64^Cu]Cu-DOTAGA-ch14.18/CHO in CHP-134 (NB) or HEK-293 (ctrl) bearing mice, and [^64^Cu]Cu-DOTAGA-4G7SDIE accumulation in CHP-134 (NB) bearing mice. (**b**-**c**) %ID/cc tumor and PET images of [^64^Cu]Cu-DOTAGA-ch14.18/CHO injected mice with and without blocking (adapted from reference 3)

Schmitt et al. described preparation and evaluation of Cu-64-labeled chimeric antibody ch14.18/CHO [33], which is clinically approved immune-therapeutics for the treatment of neuroblastoma that specifically targets tumor-associated antigen disialoganglioside GD2 highly expressed in neuroblastoma. For in vivo evaluation of tumor accumulation and imaging, Cu-64 was labeled to ch14.18/CHO using metal chelator DOTAGA ([^64^Cu]Cu-DOTAGA-ch14.18/CHO). When injected into disialoganglioside GD2 expressing CHP-134 tumor-bearing mice, [^64^Cu]Cu-DOTAGA-ch14.18/CHO showed its maximal accumulation in tumor at 48 h post-injection (14.2 ± 3.4%ID/cc), while its uptake was significantly reduced with using control HEK-293-bearing mouse or Cu-64-labeled control antibody (CD19-targeting 4G7SDIE) (Fig. 1a). Furthermore, disialoganglioside GD2-mediated uptake of [^64^Cu]Cu-DOTAGA-ch14.18/CHO was verified by receptor-blocking experiment with significantly lower accumulation of the tracer when unlabeled access ch14.18/CHO was injected (Fig. 1b, c) validating the tracer as a promising diagnostic radiopharmaceutical for guiding immunotherapy of neuroblastoma.

Since CD-30 is highly expressed in malignant lymphoma such as Hodgkin’s lymphoma and anaplastic large cell lymphoma, and the therapeutic regimen depends on the confirmation of receptor expression, it is critical to validate CD-30 expression. Tissue biopsy followed by immunohistochemistry is the current method to check the receptor expression, but it is invasive and limited to local tumors [34]. Furthermore, although there is a targeted therapeutic option for CD-30 expressing lymphoma utilizing antibody–drug-conjugate (Brentuximab vedotin), high relapse percentage and side effects such as febrile neutropenia urgently require development of better therapeutic option. To tackle above mentioned issues, Yang et al. developed chimeric monoclonal antibody (IMB16) targeting CD-30 glycosylated receptor protein and its Cu-64-labeled version using chelator NOTA-conjugated IMB16 ([^64^Cu]Cu-NOTA-IMB16) for non-invasive diagnosis of CD-30 expressing lymphoma and potential radio-immunotherapeutic regimen using Lu-177 instead of Cu-64 [34]. It was found that [^64^Cu]Cu-NOTA-IMB16 accumulated in CD-30 expressing Karpas299 tumor, while minimal accumulation was observed in CD-30 negative Raji tumor, in mouse model. Moreover, when CD-30 was blocked by injection of access IMB16, uptake of [^64^Cu]Cu-NOTA-IMB16 in Karpas299 tumor was reduced significantly confirming receptor-mediated accumulation of the radio-tracer demonstrating great potential of [^64^Cu]Cu-NOTA-IMB16 as a non-invasive tool for diagnosis of CD-30 expressing lymphoma.

Nectin4 is a cell adhesion molecule, which controls cell-to-cell adhesion process that is critical for maintenance of tissue functions and development of organs [35]. It is expressed in normal tissues such as lung, placenta, and skin with low level, while cancers including breast, lung, gastric, and bladder carcinomas overexpress nectin4, which regulates tumor progression and metastatic ability leading to poor prognosis in patients [36, 37]. Enfortumab vedotin (Padcev®) is a nectin4-targeting FDA-approved antibody drug conjugate against urothelial carcinoma. Although therapeutic efficacy of Padcev® has been already clinically proved, its biodistribution data as well as a method for identifying patients with nectin4 expression level and providing the information about likely patients who would respond to Padcev® treatment have not been developed. To provide such information, Huang et al. radiolabeled Padcev® with Cu-64 using NOTA ([^64^Cu]Cu-NOTA-Padcev) and evaluated its in vivo nectin4-expressing tumor imaging capability [38]. They showed [^64^Cu]Cu-NOTA-Padcev clearly visualized nectin4-positive NCI-N87 tumor, while the tumor uptake of [^64^Cu]Cu-NOTA-Padcev was minimal in nectin4-negative HGC-27 tumor and nectin4-blocked NCI-N87 tumor facilitating its potential as a non-invasive diagnostic tool for selecting patients for Padcev® treatment.

**Fig. 2:**
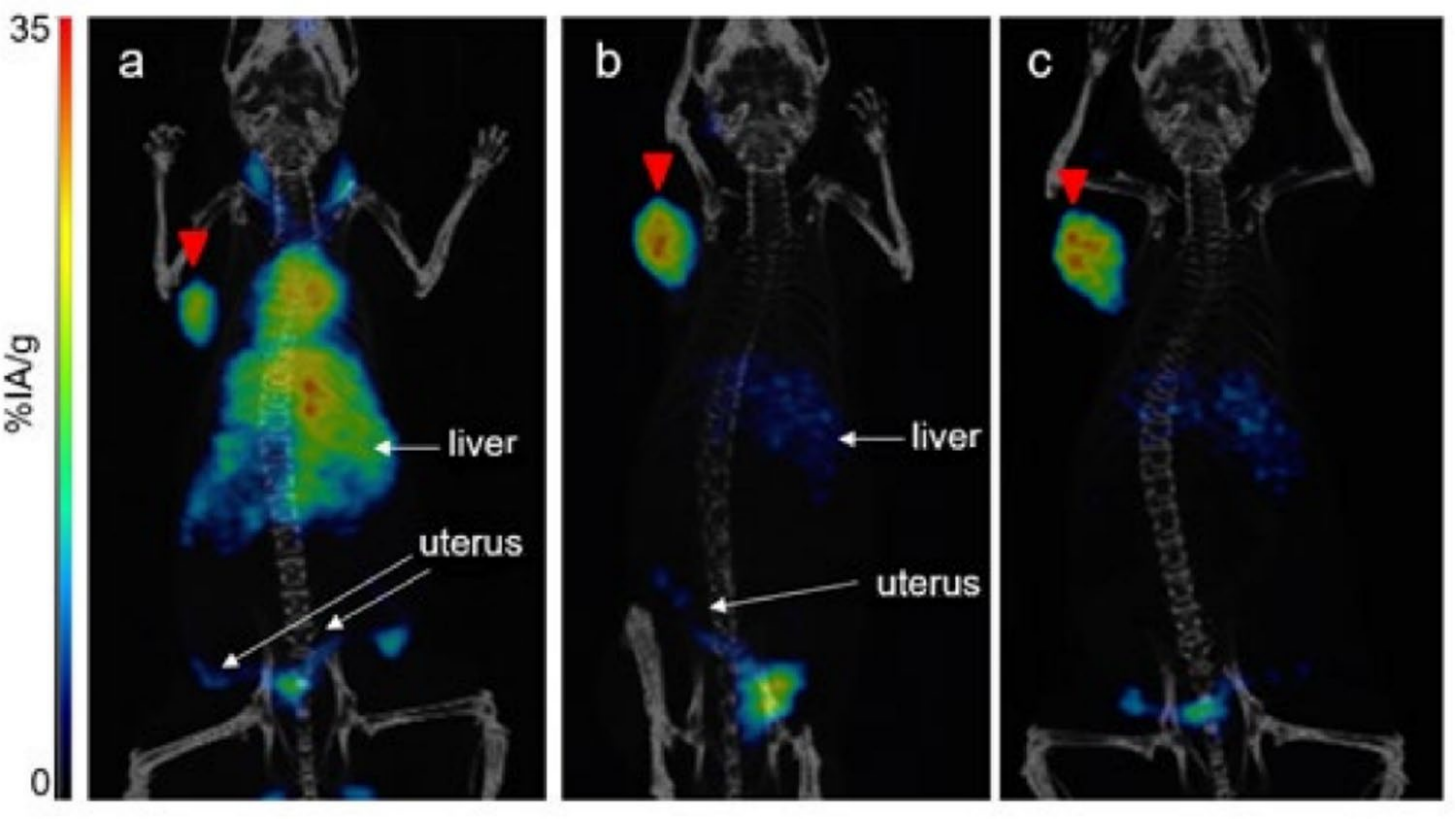
PET images of TEM-1 expressing SK-N-AS tumor-bearing mouse at (**a**) 4 h, (**b**) 24 h, and (**c**) 48 h post-injection of [^64^Cu]Cu-DOTA-1C1m-Fc (adapted from reference 10)

Although full length antibodies can be apodictically exploited as an efficient tool for delivering radionuclides to tumor site, its size (~ 150 kDa) prevents it from being excreted by glomerular filtration resulting in slow blood clearance leading to suboptimal tumor-to-background ratio [29]. To overcome the above-mentioned disadvantage, engineered antibody fragments or fusion proteins such as fragment antigen-binding region (Fab) and single-chain variable fragment (scFv) with faster clearance from the circulation have been applied to nuclear imaging for cancer diagnosis. Furthermore, engineered fragments and fusion proteins may offer improved tumor penetration as well as homogenous tissue distribution compared to full length antibodies [29]. Delage et al. recently developed fusion protein antibody that specifically binds to tumor endothelial marker 1 (TEM-1), which is highly expressed in tumor stroma and vessels in various cancers. Their fusion protein antibody (1C1m-Fc) was conjugated with DOTA, labeled with Cu-64, and evaluated for its tumor imaging potential [39]. TEM-1 positive tumor was already visible at 4 h post-injection and [^64^Cu]Cu-DOTA-1C1m-Fc was almost completely cleared from other organs thereafter (Fig. 2), verifying its potential as a new cancer imaging radiopharmaceutical.

Despite the fact that antibody fragment is a useful tool for radionuclide delivery to tumor site, it still suffers from prolonged retention in kidneys interfering with clear visualization of cancer. This issue was tackled by Suzuki et al. by utilizing enzymes present on brush border membrane in proximal renal tubule, which recognize amino acid sequence Met-Val-Lys (MVK) and cleave the linkage between Met and Val [40]. Subsequently, radiolabeled construct is removed from the kidneys by urinary excretion. They prepared Cu-64-labeled NOTA-MVK-Fab ([^64^Cu]Cu-NOTA-MVK-Fab) and compared its tumor accumulation and renal excretion with [^64^Cu]Cu-NOTA-Fab, in which the linkage between NOTA and Fab is a thiourea bond, using MC-38 bearing mouse model [41]. It was found that the injection of [^64^Cu]Cu-NOTA-MVK-Fab resulted in significantly lower kidney radioactivity compared to [^64^Cu]Cu-NOTA-Fab facilitating enzymatic strategy to reduce kidney radioactivity was feasible to produce imaging radiopharmaceuticals with increased tumor-to-background ratio.

CD-38 is a transmembrane glycoprotein that is overexpressed in malignant plasma cells such as multiple myeloma [42], and it is an attractive receptor for targeted therapy. Daratumumab is a IgG1 monoclonal antibody against CD-38 and it has shown promising therapeutic efficacy in relapsed/refractory multiple myeloma patients [43]. Current method of confirming CD-38 expression is to use bone marrow biopsy samples, but it is invasive as well as prone to yielding false negative results. Non-invasive nuclear imaging to detect CD-38 using Zr-89-labeled full length daratumumab has been studied, but poor tumor contrast images were produced due to long-lasting blood radioactivity caused by full length antibody [44]. Kang et al. prepared an antibody fragment (F(ab’)_2_) of daratumumab by removing Fc region and evaluated its CD-38 targeting ability. They radiolabeled Cu-64 to F(ab’)_2_ using NOTA ([^64^Cu]Cu-NOTA-Dara-F(ab’)_2_) and obtained PET images of CD-38 expressing Ramos tumor mouse model [45]. It was found that [^64^Cu]Cu-NOTA-Dara-F(ab’)_2_ bound to the tumor CD-38 receptor mediated manner and produced high tumor contrast PET images validating improved performance of radiolabeled antibody fragments as an diagnostic radiopharmaceuticals compared to full length antibodies.

Another example of utilizing antibody fragment F(ab’)_2_ as a delivery vehicle for nuclear imaging was demonstrated by Huang et al. Triple negative breast cancer (TNBC) is a refractory cancer with limited treatment options such as surgery, chemotherapy, and radiation therapy, and significant number of TNBC patients have advanced diseases and are not even eligible for surgery necessitating the development of precise diagnostic method [46]. Trophoblast cell surface antigen 2 (Trop2) is associated with tissue formation and organ development and naturally expressed in normal cells. However, Trop2 expression is highly upregulated in cancers, including TBNC, rendering their proliferating ability and metastatic potential [47]. Sacituzumab govitecan (Trodelvy™) is an antibody drug conjugate developed for targeting Trop2, and its therapeutic efficacy has been impressive. However, the visualization of the drug and identification of patients who are likely responding positively are still required. Huan et al. prepared F(ab’)_2_ from Trodelvy (Trodelvy-F(ab’)_2_) and radiolabeled it with Cu-64 using NOTA ([^64^Cu]Cu-NOTA-Trodelvy-F(ab’)_2_) for the evaluation of its Trop2 targeting ability [48]. It was demonstrated that [^64^Cu]Cu-NOTA-Trodelvy-F(ab’)_2_ significantly accumulated in Trop2-expressing (MDA-MB-468) mouse model, while minimal accumulation was observed in Trop2-negative (MDST8) mouse model and blocking group suggesting receptor-mediated specific uptake. Furthermore, [^64^Cu]Cu-NOTA-Trodelvy-F(ab’)_2_ generated more contrasting tumor images compared to [^64^Cu]Cu-NOTA-Trodelvy due to faster clearance of the antibody fragment from the circulation displaying potential usage in Trop2 imaging.

### Zr-89

Zr-89 has attracted a lot of attention from the field of nuclear imaging due to its ideal half-life (78.4 h), which matches with biological half-life of typical antibodies, and relatively low positron energy (E_mean_ = 395.5 keV) that can produce PET images with high resolution [49, 50], and there have been vast number of (pre)clinical studies utilizing Zr-89 as a PET imaging radionuclide. Ameloblastoma is an odontogenic carcinoma, which shows locally aggressive and destructive behavior while it is benign, and since there is no molecular marker for ameloblastoma, it is difficult to determine surgical margin resulting in high recurrence rate [51]. It has been known that ameloblastoma expresses epidermal growth factor receptor (EGFR) in the majority of clinical cases [52, 53], panitumumab, FDA-approved EGFR-targeting monoclonal antibody, is a promising candidate that can be radiolabeled with imaging radionuclide providing better assessment about surgical margin for ameloblastoma. Stone et al. prepared Zr-89-labeled panitumumab using DFO as a chelator ([^89^Zr]Zr-panitumumab) and evaluated its ameloblastoma imaging potential using patient derived xenograft (PDX) model [8]. They showed that [^89^Zr]Zr-panitumumab accumulated well in ameloblastoma PDX, while blocking EGFR with access administration of non-labeled panitumumab significantly reduced the uptake, demonstrating potential application of [^89^Zr]Zr-panitumumab as a non-invasive molecular probe that can provide useful information about surgical margin for ameloblastoma.

**Fig. 3:**
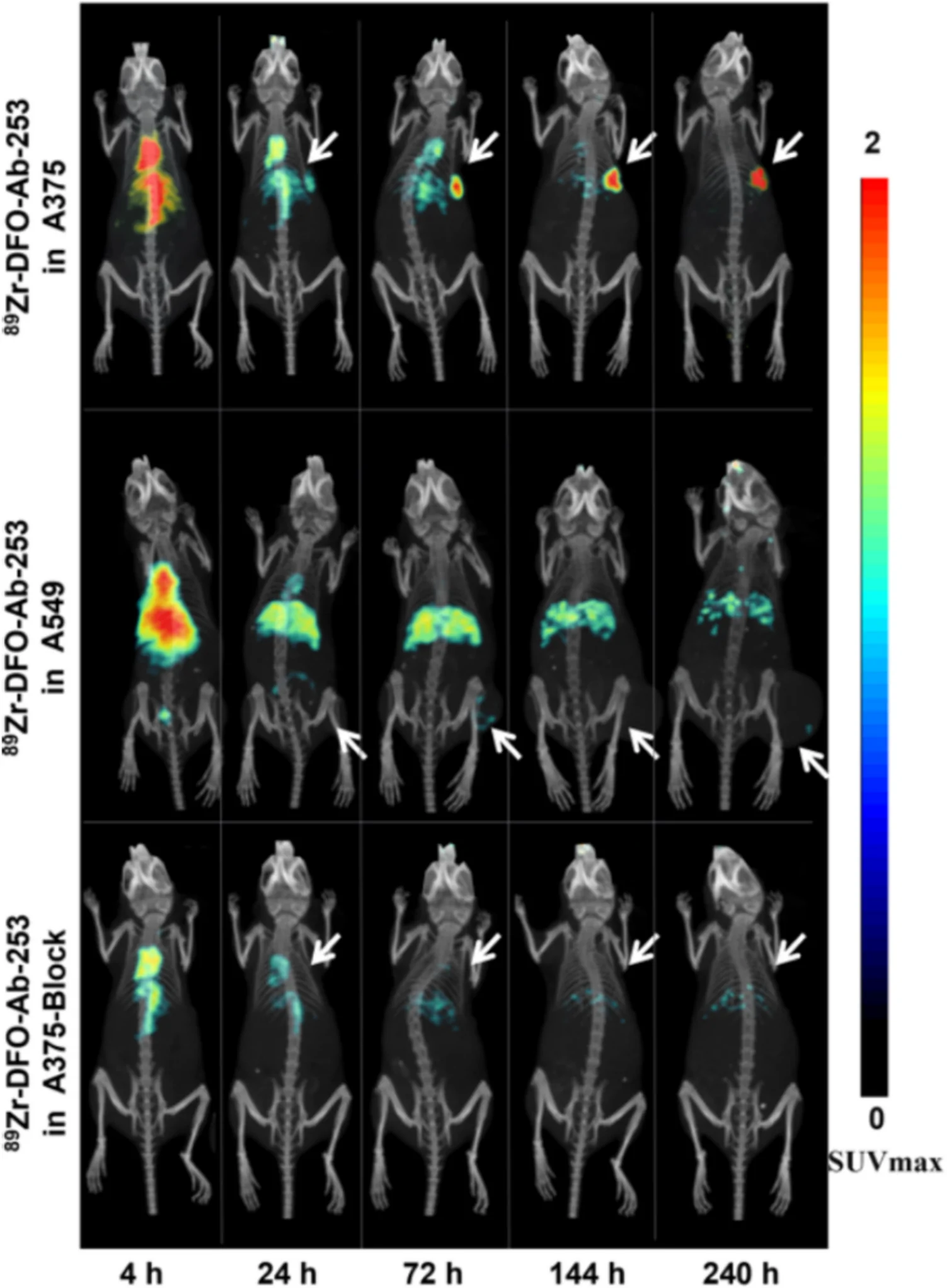
PET images of CD146-positive A375-bearing and CD146-negative A549-bearing mouse models administered with [^89^Zr]Zr-DFO-Ab253 (adapted with permission from Guo X, Hu M, Zhang Q, Liu J, Shi J, Tang Y, et al. Preclinical ImmunoPET Imaging Using a Zr-89-Labeled Anti-CD146 Monoclonal Antibody for Diagnosis of Melanoma. Mol Pharm. 2024;21:4490–7. Copyright 2024 American Chemical Society)

 Melanoma is skin cancer originated from melanocytes, and it is characterized by aggressive proliferation and high metastatic potential [54]. Its diagnosis depends on the clinical symptoms and histological features such as Breslow thickness, but those diagnostic methods are not able to present the whole tumor burden resulting in poor prognosis [55, 56]. CD146 is a cell adhesion molecule, which exists on the tumor vascular endothelial cells, and it is known that CD146 is highly expressed in primary and metastatic melanomas [57]. Ab253 is a monoclonal antibody against CD146, and Guo et al. evaluated its CD146-expressing melanoma imaging using Zr-89 as an imaging radionuclide. They synthesized Zr-89-labeled Ab253 using a chelator DFO ([^89^Zr]Zr-DFO-Ab253) and assessed its tumor targeting in melanoma-bearing mouse model [58]. It was found that [^89^Zr]Zr-DFO-Ab253 was able to accumulate and detect CD146-expressing melanoma (A375 cell), while there was minimal uptake in CD146-negative tumor (A549 cell). Furthermore, when CD146 was blocked in CD146-positive tumor model using the administration of access unlabeled Ab253, [^89^Zr]Zr-DFO-Ab253 uptake was also minimal demonstrating receptor-mediated uptake of the radiotracer (Fig. 3) facilitating great potential of [^89^Zr]Zr-DFO-Ab253 as a molecular probe that can non-invasively detect CD146-expressing melanomas.

**Fig. 4:**
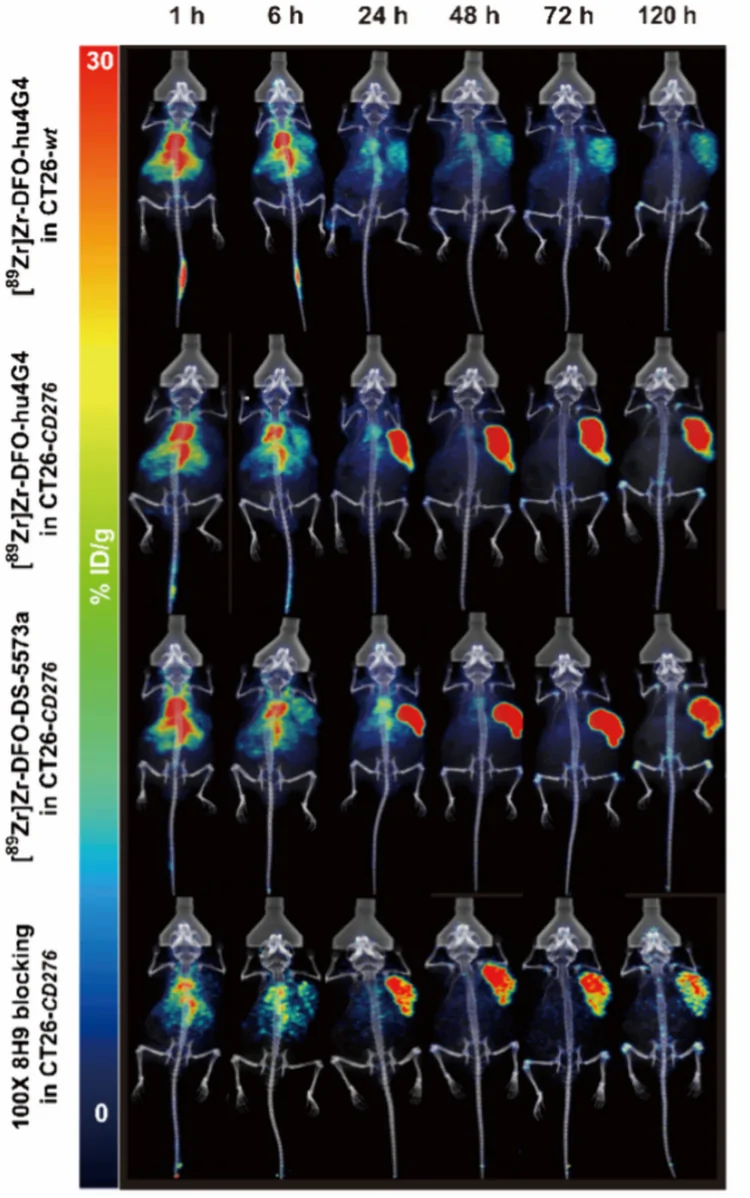
PET images of B7-H3-positive CT26-CD276-bearing mouse administered with [^89^Zr]Zr-DFO-hu4G4 or [^89^Zr]Zr-DFO-DS-5573a. [^89^Zr]Zr-DFO-hu4G4 injected B7-H3-negative (CT26-wt) tumor and B7-H3-blocked model are also shown (adapted with permission from Zheng M, Liu Q, Zhang H, Wang Y, Zhang K, Mu H, et al. Development of a Specifically Labeled (89)Zr Antibody for the Noninvasive Imaging of Tumors Overexpressing B7-H3. Mol Pharm. 2024;21:5205–16. Copyright 2024 American Chemical Society)

B7-H3, also known as CD276, is a transmembrane glycoprotein that act as an immunomodulator [59]. B7-H3 is highly expressed in some solid tumors as well as hematologic carcinomas, and it promotes cancer cell proliferation, regulates tumor angiogenesis, and renders cancer resistance against various cancer therapeutics including chemotherapeutics [60, 61]. Zhen et al. recently developed a monoclonal antibody against B7-H3 (hu4G4) to facilitate accurate diagnosis and treatment with high efficacy through proper patient selection. They produced Zr-89-labeled hu4G4 using DFO ([^89^Zr]Zr-DFO-hu4G4) and evaluated its targeting and imaging capability in B7-H3 expressing cancer model [62]. It was shown that [^89^Zr]Zr-DFO-hu4G4 specifically accumulated in B7-H3 expressing tumor in vivo, and the result was comparable to that of commercially available antibody against B7-H3 (DS-5573a) (Fig. 4). Furthermore, the tumor uptake of [^89^Zr]Zr-DFO-hu4G4 was negligible when B7-H3 was blocked by addition of excess amount of the antibody proving receptor-mediated uptake of [^89^Zr]Zr-DFO-hu4G4 potentiating its usage as a B7-H3 expressing tumor targeting radiotracer.

Multiple myeloma (MM) is a cancer originated from plasma cells in bone marrow causing osteolytic bone lesions, which are refractory and relapsed even after radiation therapy and immunoregulatory treatment [63, 64]. B-cell maturation antigen (BCMA) a tumor necrosis factor that is overexpressed in malignant plasma cells, and BCMA has been the promising target for targeted therapeutics including ADC and chimeric antigen receptor (CAR)-T cells [65]. However, due to heterogeneous nature of BCMA expression in MM, it is critical to have diagnostic methods for proper patient selection, who is likely to respond to BCMA-targeted therapy, and monitoring of disease progression [66]. Recently, Ma et al. evaluated radiolabeled version of BCMA-targeting monoclonal antibody as a diagnostic radiopharmaceutical for detecting BCMA-positive MM [67]. They prepared Zr-89-labeled BCMA-targeting antibody ([^89^Zr]Zr-DFO-BCMAh230430) and showed the radiotracer accumulated in BCMA-positive MM.1S tumor in vivo (7.7%ID/g at 168 h post-injection) displaying the potential of [^89^Zr]Zr-DFO-BCMAh230430 as a non-invasive diagnostic tool for MM.

Trophoblast cell surface antigen 2 (Trop2) is an antigen that is naturally expressed in normal cells and controls embryogenesis, tissue formation and organ development [68]. However, trop2 is overexpressed in some solid tumors including gastrointestinal, breast, and prostate cancers rendering high proliferation rate and metastatic potential leading to poor prognosis [47]. Sacituzumab govitecan (Trodelvy) is an ADC targeting trop2, and it has been shown a great clinical efficacy against triple negative breast cancer [69]. Nevertheless, information about the biodistribution of Trodelvy and precise determination of beneficiaries who are likely to respond positively upon Trodelvy treatment are difficult to decide. Huang et al. radiolabeled Trodelvy with Zr-89 and evaluated its tumor targeting in trop2-expressing NCI-N87 and MDA-MB-468 cancers in vivo [70]. They showed Zr-89-labeled Trodelvy ([^89^Zr]Zr-DFO-Trodelvy) accumulated well in tumors (14.3 and 10.0%ID/g at 48 h post-injection for NCI-N87 and MDA-MB-468 cancers, respectively) proving a potential of [^89^Zr]Zr-DFO-Trodelvy as a non-invasive imaging molecular probe for trop2-expressing cancers.

Epidermal growth factor receptor 1 (EGFR) governs cell growth and progression by binding its ligand such as epiderma growth factor and amphiregulin [71], and its overexpression in some tumors render aggressiveness [72]. Quantification of EGFR expression using biopsy is not accurate and difficult due to the heterogeneous nature of EGFR expression in cancers and the changes in the expression level over time [73, 74]. Binding domain of EGFR is composed of 4 units, and domain III binding therapeutic antibodies such as cetuximab and panitumumab have been radiolabeled using imaging radionuclides and tested as patient selecting probe [75, 76]. However, EGFR-targeting radiolabeled antibodies cannot provide information about response to the therapeutic antibody because binding site competition between radiolabeled probe and therapeutics necessitating development of probe binding to different domain of EGFR. Recently, EGFR domain II binding antibody fragment was developed (8709-scFv-Fc) and its radiolabeled version ([^89^Zr]Zr-8709-scFv-Fc) was evaluated for its tumor uptake in vivo by Alizadeh et al. [77]. It was found that [^89^Zr]Zr-8709-scFv-Fc accumulated in EGFR-expressing DLD-1 tumor and its uptake was not altered by the administration of EGFR-binding antibody (nimotuzumab) showing great potential of the radiotracer as a probe for monitoring response to antibody therapy against EGFR-expressing cancers.

Human epidermal growth factor receptor 2 (HER2) is a receptor tyrosine kinase that is naturally expressed in breast cell regulating normal growth [78]. However, 15–25% of all breast cancer overexpress HER2 and activate intracellular pathways including mitogen activated protein kinase (MAPK) and phosphoinositide 3 kinase (PI3K) leading to uncontrolled cell proliferation and anti-apoptosis resulting in poor prognosis [78–80]. There have been many therapeutic antibodies targeting HER2 such as trastuzumab and pertuzumab, which binds to domain IV and II, respectively, and the radiolabeled versions of the antibodies for the diagnosis of HER2-expressing cancer. However, radiolabeled full-length antibodies against HER2 cannot be utilized in monitoring efficacy of the therapeutic antibody or disease progression due to the competition for the same binding site. Nanobody is an antigen binding fragments having size approximately 15 kDa, and it is known that nanobody has a more rapid diffusion in vivo and better tissue penetration compared to full-length antibody [81]. HER2 domain I binding nanobody, 2Rs15d, has been developed and Ducharme et al. prepared its Zr-89-labeled version ([^89^Zr]Zr-DFO-2Rs15d) and evaluated HER2-expressing cancer targeting in vivo [82]. They showed [^89^Zr]Zr-DFO-2Rs15d specifically accumulated in HER2-expressing tumor (BT-474), while the radiotracer had minimal uptake in HER2-negative tumor (MDA-MB-468), displaying potential of [^89^Zr]Zr-DFO-2Rs15d as a non-invasive molecular probe for monitoring antibody therapeutic efficacy in HER2-expressing cancers.

### I-124

I-124 with half-life of 4.2 days and dual positron energies of 1532 and 2135 keV is not an ideal radionuclide for PET since the positrons with relatively high energies would travel long distance before annihilation with electron resulting in deceitfully identified line of response, and thereby, lower resolution [83]. However, relatively longer half-life of I-124 compared to other positron emitters and direct radiolabeling method using tyrosine residues in an antibody or antibody fragments without need for chelating agents make it attractive radionuclide for PET. CD147 is a glycoprotein, which acts as a regulator of matrix metalloproteinase [84]. It is known that CD147 is minimally expressed in normal cells, but highly expressed in some tumors, making it a valuable target for cancer therapy [85]. There have been a couple of CD147-targeted therapeutic methods such as CD147/GPC3 chimeric antigen receptor (CAR) cell therapy, and it is necessary to develop accurate CD147 detection strategies as CD147-targeted therapeutics continue to evolve. Ma et al. synthesized I-124-labeled anti-CD147 antibody ([^124^I]I-anti-CD147) and evaluated its CD147-targeting efficiency in vivo [86]. It was demonstrated that [^124^I]I-anti-CD147 was specifically accumulated in CD147-expressing LS174T tumor (SUV_max_ at 48 h post-injection was 1.78), while the accumulation was minimal in CD147-negative BGC823 tumor (SUV_max_ at 48 h post-injection was 0.51) showing a great potential of [^124^I]I-anti-CD147 as a radiotracer for CD147-expressing cancers.

Programmed cell death protein-1 (PD-1) is a cell surface antigen expressed on T-cell, and it controls T-cell inflammatory activity resulting in induction of immune tolerance and prevent autoimmune diseases [87]. However, PD-1 is also involved in the immune escape of cancer by binding to programmed cell death 1 ligand 1 (PD-L1), which is expressed on cancer cells, and making T-cell not to recognize cancer cells [88]. PD-L1 has been studied as a target for therapy using immune checkpoint inhibitor (ICI), but presence of PD-L1 does not necessarily reflect or predict therapeutic outcome as patients with no PD-L1 detection still benefit from ICI treatment [89, 90]. PD-L2 induces immune escape for cancer with the same mechanism as PD-L1, and it has been known that some tumors highly express PD-L2 [91]. Recently, Yao et al. produced a bispecific antibody (NB12) that simultaneously binds to PD-L1 and PD-L2 to address the discrepancy between cell death ligand detection and therapeutic efficacy of ICI. They radiolabeled NB12 with I-124 ([^124^I]I-NB12) and evaluated its accumulation in PD-L1 or PD-L2 expression tumors in vivo [92]. It was found that [^124^I]I-NB12 specifically accumulated in PD-L1 or PD-L2 expressing A549 tumors, while there was negligible accumulation of [^124^I]I-NB12 in tumors with no PD-L1 or PD-L2 expression demonstrating a great potential of the radiotracer as a diagnostic tool that can provide more vivid information for ICI.

## Antibody-Based Radiopharmaceuticals for Cancer Therapy

### Lu-177

**Fig. 5:**
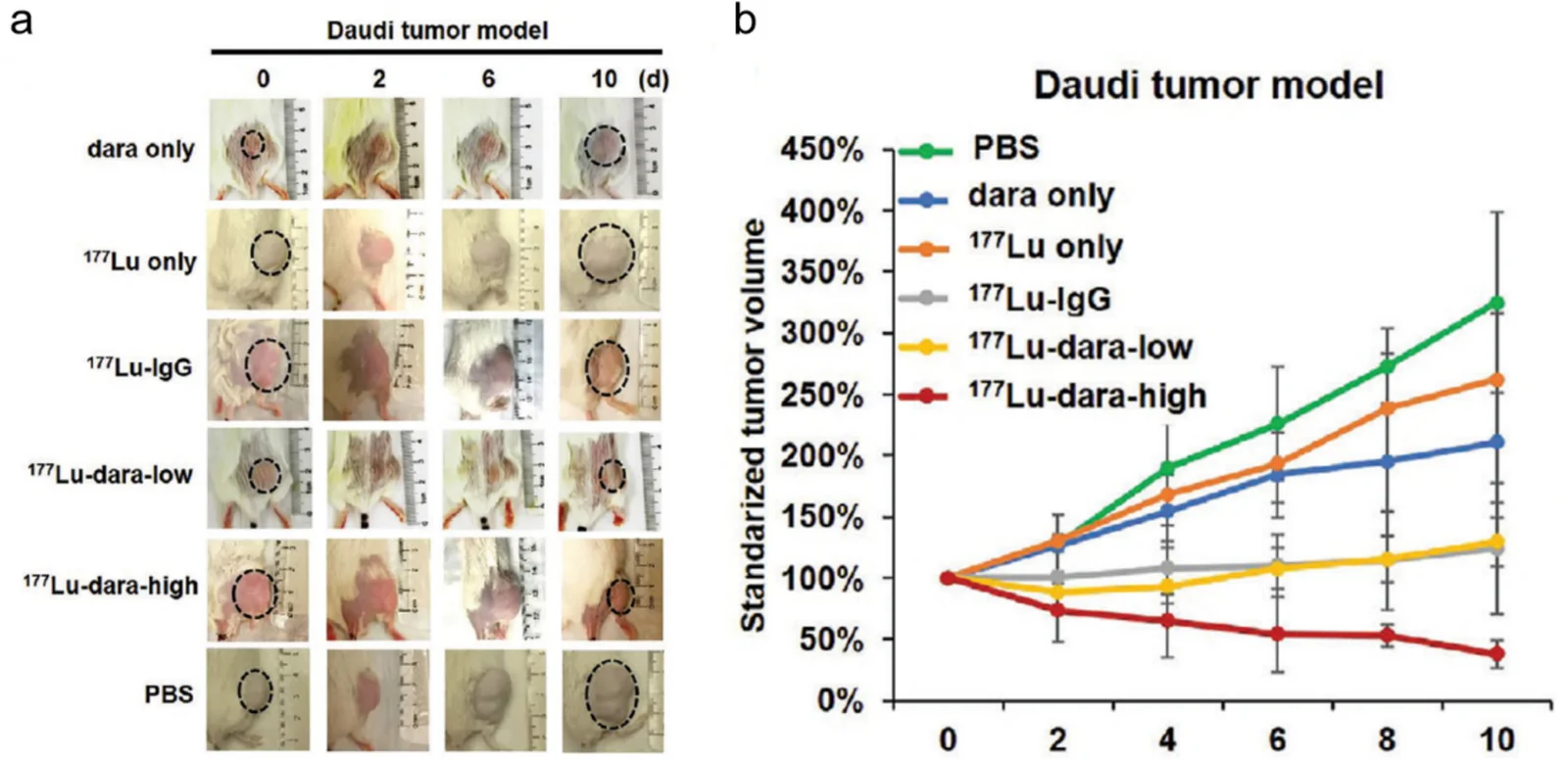
Therapeutic efficacy of [^177^Lu]Lu-dara in Daudi tumor model (**a**) representative photograph of tumor site (**b**) standardized tumor volume (adapted from reference 70)

Lu-177 has medium β^−^ energy of 490 keV and relatively short tissue penetrating range of less than 2 mm that are characteristics of a radionuclide suitable for the development of targeted radionuclide therapy (TRT), and it has been shown that Lu-177-labeled peptides are effective for the treatment of micro-metastases due to high radiation absorbed dose in tumor [93, 94]. Due to its long half-life (6.73 days), which matches with biological half-lives of typical antibody, Lu-177 has been radiolabeled to antibodies that specifically target different types of cancers. Multiple myeloma (MM), a subtype of lymphoma, is hematologic cancer with high incidence [95]. Immunotherapy and some proteasome inhibitors have shown effectiveness, but MM eventually develops drug resistance and becomes refractory necessitating development of more effective therapeutic options [96]. Since malignant plasma cells outside bone marrow are radiosensitive, targeted radionuclide therapy against MM is a reasonable approach for high therapeutic efficacy [97]. CD38, which is a glycoprotein receptor involved in cytoplasmic calcium metabolism, is highly expressed in malignant plasma cells making it plausible therapeutic target [43]. Daratumumab (Dara) is a monoclonal antibody that specifically binds to CD38, and it has been approved by FDA for the treatment of relapsed MM. Kang et al. synthesized Lu-177-labeled Dara using DTPA ([^177^Lu]Lu-dara) and evaluated its therapeutic efficacy in vivo [98]. It was found that [^177^Lu]Lu-dara caused significant tumor suppression in CD-38 expressing Daudi tumor model validating [^177^Lu]Lu-dara as a promising targeted radionuclide therapeutic agent for the treatment of CD-38 expressing malignant plasma cells (Fig. 5).

Epithelial ovarian carcinoma (EOC) is normally diagnosed at advanced stage resulting in highest mortality among gynecologic cancers [99]. YKL40 is a glycoprotein that regulates proliferation, differentiation, and angiogenesis, and it is known that YKL40 is highly expressed in some malignant tumors [100]. Preclinical investigations have shown that YKL40 inhibition could be a potential therapeutic target and YKL40-neutralizing antibody has shown therapeutic effects in preclinical studies [101]. To exploit YKL40 as a subject for targeted radionuclide therapy, Chang et al. prepared Lu-177-labeled YKL40 antibody using DTPA ([^177^Lu]Lu-DTPA-YKL40 Ab) and evaluated its therapeutic efficacy in YKL40-expressing tumor model [102]. It was demonstrated that [^177^Lu]Lu-DTPA-YKL40 Ab resulted in significant inhibition of tumor growth compared to non-treated control potentiating [^177^Lu]Lu-DTPA-YKL40 Ab as a YKL40-targeting therapeutic radiopharmaceutical.

**Fig. 6:**
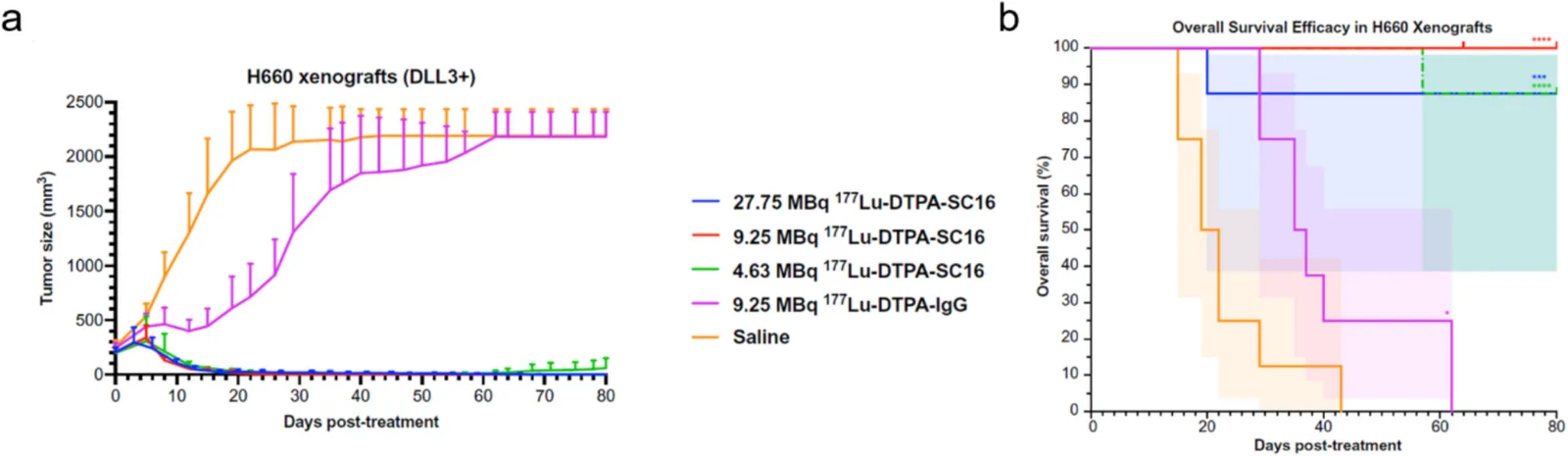
Therapeutic efficacy of [^177^Lu]Lu-DTPA-SC16 in H660-bearing mouse model (**a**) tumor volume changes and (**b**) overall survival (adapted from reference 81)

Androgen receptor signaling in prostate cancer (PC) is important for tumor cell survival, and it has been shown that androgen receptor signaling inhibitors (ARSIs) such as abiraterone and enzalutamide are effective in managing PC. However, ARSIs are not curative, and tumors eventually develop resistance, aggressiveness, and metastases leading to patient’s death [103]. The resistance is caused by lineage plasticity from AR-dependent PC to AR-independent neuroendocrine PC (NEPC), which is characterized by the loss of tumor suppressor genes such as TP53 and RB1 [104, 105]. Delta-like ligand 3 (DLL3) has been utilized as a therapeutic target for NEPC and small-cell lung cancer (SCLC) due to its selective expression compared to normal cells [106, 107], and ADC targeting DLL3 (rovalpituzumab-tesirine) has been clinically evaluated for the treatment of DLL3-expressing NEPC, but it was withdrawn due to toxicity issue [108]. Korsen et al. took an advantage of the monoclonal antibody (SC16) from the ADC for its DLL3-targeting, radiolabeled with Lu-177 using CHA-A”-DTPA ([^177^Lu]Lu-DTPA-SC16), and evaluated therapeutic efficacy of the radiopharmaceutical [109]. They showed [^177^Lu]Lu-DTPA-SC16 effectively inhibited tumor growth using DLL3-expressing H660 xenograft model and the overall survival was significantly increased compared to non-treated control group (Fig. 6). Furthermore, it was found recurring NEPC was completely managed by repeating treatment of [^177^Lu]Lu-DTPA-SC16 demonstrating a great potential of the radiopharmaceutical for the treatment of NEPC.

Non-Hodgkin lymphoma (NHL) is a hematologic malignancy that highly expresses CD20. Combinational therapy using chemotherapeutics and chimeric antibody rituximab-based CD20-targeting immunotherapy has been standard care for NHL [110]. However, due to the concern of immune reaction, only single therapeutic dose has been approved and NHL often relapses and becomes refractory necessitating the development of more effective therapeutic strategies. Ofatumumab is a third-generation anti-CD20 antibody, which is fully humanized and internalizes when bound to CD20 [111], and it has been shown CD20-expressing tumors can be visualized well using radiolabeled ofatumumab [112]. Shim et al. recently prepared Lu-177-labeled ofatumumab using metal chelator CHX-A”-DTPA ([^177^Lu]Lu-ofatumumab) and evaluated its therapeutic efficacy in C20-expressing Raji xenograft [113]. They showed the treatment of [^177^Lu]Lu-ofatumumab resulted in significantly increased survival of xenograft mouse compared to non-treated group exhibiting promising potential of the radiopharmaceutical for the effective treatment of CD20-expressing NHL.

Pancreatic cancer has high level of heterogeneity, malignancy, and distant metastases leading to high lethality, and it has been shown that 5-year overall survival rate is less than 5% [114, 115]. Surgical removal is the best option for the treatment of pancreatic cancer, but it is extremely difficult to detect the cancer at an early stage, and it is not practicable to perform surgical resection for the patients who diagnosed at an advanced stage [116]. Human trophoblast cell surface antigen 2 (Trop2) is a transmembrane protein, which is overexpressed in some epithelioid cancers including pancreatic cancer. It has been shown the overexpression of Trop2 is correlated with high level of lymph node metastases leading to poor prognosis [117], making Trop2 a valuable target for pancreatic cancer diagnosis and therapy. Li et al. developed humanized antibody against Trop2 (hIMB1636), radiolabeled, and evaluated its therapeutic potential [118]. They synthesized Lu-177-labeled hIMB1636 using a chelator DOTA ([^177^Lu]Lu-DOTA-hIMB1636) and showed the administration of [^177^Lu]Lu-DOTA-hIMB1636 in Trop2-expressing T3M-4 bearing mouse resulted in significant inhibition of tumor growth compared to non-treated group. Furthermore, there was not [^177^Lu]Lu-DOTA-hIMB1636 treatment-related normal tissue toxicity displaying a great potential of [^177^Lu]Lu-DOTA-hIMB1636 as a therapeutic radiopharmaceutical in pancreatic cancer therapy.

**Fig. 7:**
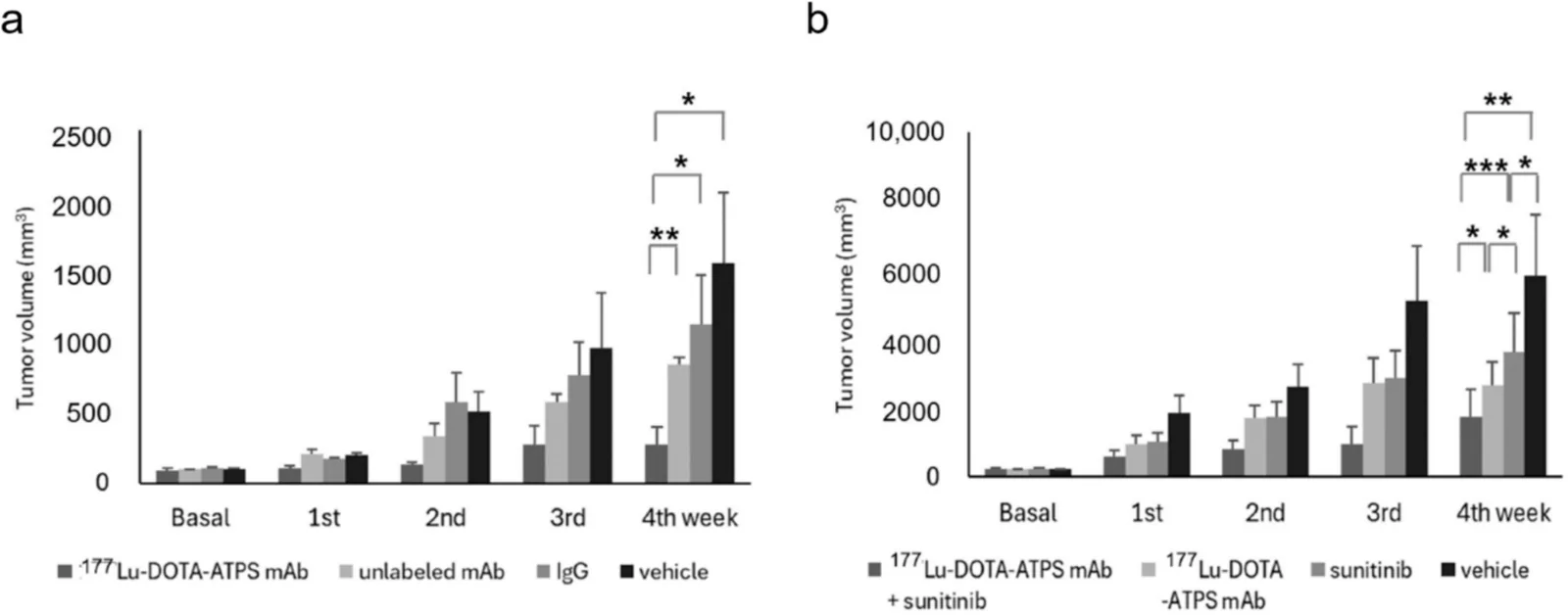
Therapeutic efficacy of [^177^Lu]Lu-DOTA-ATPS mAb in MKN-45-bearing mouse model tumor volume changes when treated with (**a**) [^177^Lu]Lu-DOTA-ATPS mAb, unlabeled mAb, or IgG, and (**b**) [^177^Lu]Lu-DOTA-ATPS mAb + sunitinib, [^177^Lu]Lu-DOTA-ATPS mAb, sunitinib (adapted from reference 96)

Angiogenesis is the formation of a new blood vessel, and it is a critical process for normal tissue formation as well as tumor expansion, in which growth and metastasis are highly dependent on angiogenesis [119]. Adenosine triphosphate synthase (ATPS), which is responsible for the synthesis of ATP, is normally resides in the inner membrane of mitochondria, but it is also found on the surface of endothelial and tumor cells (ectopic ATPS) [120]. Antibodies, which specifically bind to ATPS, have been developed, it was demonstrated that the antibody-binding hindered the activity of ATPS and inhibited tumor proliferation [121, 122], and it was also shown that Zr-89-labeled anti-ATPS antibody selectively accumulated in ATPS-expressing tumor model in vivo [123]. Park et al. recently exploited ATPS-binding monoclonal antibody as a delivery molecule for a therapeutic radionuclide. They prepared Lu-177-labeled ATPS-binding antibody using DOTA ([^177^Lu]Lu-DOTA-ATPS mAb) and evaluated its therapeutic efficacy in ATPS-expressing MKN-45 bearing mouse [124]. Treatment of [^177^Lu]Lu-DOTA-ATPS mAb resulted in significant reduction of tumor growth and reinforced the effects of chemotherapeutic sunitinib, which is a receptor tyrosine kinase inhibitor, confirming a potential of radioimmunotherapy targeting ATPS (Fig. 7).

### Ac-225

Targeted radionuclide therapy (TRT) has drawn a lot of attention due to the successful clinical outcome of Lu-177-labeled peptides targeting somatostatin receptor (SSTR) or prostate specific membrane antigen (PSMA) and following marketing authorization of the radiolabeled agents, e.g. Lutathera®. However, therapeutic efficacy using Lu-177-based radiopharmaceutical is somewhat limited because long radiation range of β^−^ emission makes the large portion of energy deposited in the surrounding normal tissues rather than concentrated in the tumor area [125]. Recently, researchers turned their attention to alpha particle emitting radionuclides since it is known that alpha particle has the superior linear energy transfer (LET), compared to β^−^ particle or Auger electrons, leading to double stand DNA breaks [126]. Among the alpha particle emitting radionuclides, Ac-225, with half-life of 9.92 days and multiple emission of alpha particles during its decay, has been standing out due to its clinical success [127]. Since physical half-life of Ac-225 is relatively long, it is quite reasonable to develop radiopharmaceuticals using antibody as a targeting moiety.

Malignant mesothelioma an aggressive cancer, which is primarily originated from peritoneal mesothelial surfaces. Surgical removal of mesothelioma is possible only if it is diagnosed at early stages, but mesothelioma is often at advanced stages when it is first diagnosed [128, 129]. Chemotherapy is the first-line treatment for mesothelioma, but it has been shown that the overall survival duration is less than 12 months necessitating the development of more effective therapeutic methods [128, 129]. Podoplanin (PDPN) is a transmembrane glycoprotein that is overexpressed in mesothelioma, and it regulates epithelia-mesenchymal transition, invasion, migration, and metastases of the cancer leading to poor prognosis [130]. Anti-PDPN antibodies have been developed and preclinically investigated their therapeutic effects, and it has been found that the therapeutic antibodies inhibited tumor progression and metastases [131]. PDPN-targeting antibody (NZ-12) has been radiolabeled with β^−^ emitter Y-90, but complete remission was not achieved [132]. Sudo et al. radiolabeled PDPN-targeting antibody, NZ-16, which has increased binding affinity to PDPN compared to NZ-12, with Ac-225 using DOTA ([^225^Ac]Ac-NZ-16) to improve therapeutic efficacy of the antibody [133]. It was found that administration of [^225^Ac]Ac-NZ-16 in PDPN-expressing H226 xenograft resulted in significant reduction in tumor growth, and importantly there was no re-growth of the tumor, while tumors treated with [^90^Y]Y-NZ-16 re-grew demonstrating higher effectiveness of alpha particle emitters.

Prostate cancer is the most diagnosed malignancy and leading cause of death in men worldwide [114]. Standard of care for prostate cancer is surgery, radiation therapy, and androgen deprivation therapy when it is diagnosed at early stage, but significant portion of patients progresses to metastatic and castration resistant prostate cancer (mCRPC). The treatment options for mCRPC patients include chemotherapy, androgen signaling inhibition therapy, immunotherapy, and radioligand therapy ([^177^Lu]Lu-PSMA-617). However, these treatments are not curative in most of the cases and cancer progresses. PSMA is a type II glycoprotein, which is overexpressed in prostate cancer, and diagnosis for the detection of PSMA using radiopharmaceuticals such as [^68^ Ga]Ga-PSMA-11 is routinely performed. However, a lot of mCRPC patients fail to respond to PSMA-targeted therapy due to heterogeneity of PSMA expression [134, 135]. Another molecular marker discovered for mCRPC is CD46, and it was shown that its expression is highly homogeneous compared to PSMA [136]. CD46-targeting antibody was developed (YS5), and Zr-89-labeled YS5 ([^89^Zr]Zr-DFO-YS5) was preclinically evaluated for its targeting efficiency to CD46-expressing neuroendocrine prostate cancer [137]. Bidkar et al. synthesized Ac-225-labeled YS5 ([^225^Ac]Ac-DOTA-YS5) and evaluated its therapeutic efficacy in vivo [138]. It was found that treatment of [^225^Ac]Ac-DOTA-YS5 (0.25 and 0.5 μCi) resulted in significant inhibition of tumor growth compared to controls (saline and [^225^Ac]Ac-DOTA-IgG) in CD46-expressing 22Rv1 xenograft model leading to highly improved median survival of the experimental group (103 and 131 days for 0.25 and 0.5 μCi, respectively, vs. 37 and 51 days for saline and [^225^Ac]Ac-DOTA-IgG, respectively). Moreover, it was confirmed [^225^Ac]Ac-DOTA-YS5 treatment caused DNA double strand breaks in tumor tissue demonstrating promising potential of [^225^Ac]Ac-DOTA-YS5 as a therapeutic radiopharmaceutical for mCRPC.

EGFR has been a valuable target in the development of targeted therapeutics because it is overexpressed in many malignant cells, but on-target side effects have been an issue to tackle due to its expression in normal tissues [139]. To tackle the on-target toxicity issue, tumor-specific anti-EGFR antibody (mAb806) has been developed and its selective binding to tumor EGFR and EGFR.de2-7 mutant was confirmed, while the antibody does not bind to EGFR expressed on normal tissues [140, 141]. Wichmann et al. recently prepared Ac-225-labeled chimeric version of tumor-specific anti-EGFR antibody using macropa as a chelating agent ([^225^Ac]Ac-ch806), and they evaluated its therapeutic efficacy [142]. It was demonstrated that the administration of [^225^Ac]Ac-ch806 resulted in prolonged tumor suppressive effect in U87MG.de2-7 bearing mouse model leading to 100% survival of the experimental animal up to the end of study (80 days). On the other hand, median survival of [^225^Ac]Ac-ch806 or ch806-injected groups were only 9 and 29 days, respectively, showing great therapeutic potential of Ac-225-labeled tumor-specific EGFR-binding antibody.

Ovarian cancer is often diagnosed at advanced stages leading to poor survival rate around 30% [143]. Advanced ovarian cancer develops peritoneal metastases that lead to fluid accumulation in abdomen (ascites). Microscopic tumors in ascites are difficult to detect and treat, and they are the major cause of cancer recurrence [144]. Targeted alpha therapy (TAT) has attracted a lot of attention in the field of radionuclide therapy due to higher therapeutic efficacy and reduced side effects ascribe to high LET and short emission range of alpha particles, compared to β^−^ emitters. However, when full-length antibody is labeled with radionuclides having long half-life such as Ac-225, hematological toxicity and off-target side effects often become an issue limiting clinical application of TAT [145]. Pre-targeting approach in radionuclide therapy utilizes tumor targeting efficiency of antibody and fast pharmacokinetics of radioligand. In such method, antibody is injected first and radioligand is administered later, when maximum accumulate in tumor reaches while antibody in the circulation is cleared out, after which in vivo coupling between the antibody and radioligand leads to radioactivity accumulation in tumor [146]. Reaction between trans-cyclooctene (TCO) and tetrazine (Tz) belongs to inverse electron demand Diels Alder reaction (IEDDA) and is the most utilized chemistry for in vivo click chemistry [147]. MUC16 is a glycoprotein that is overexpressed in some cancers including ovarian cancer, and it is known that MUC16 promotes cancer cell proliferation and metastasis [148]. Mack et al. utilized MUC16-targeting antibody AR9.6 for pre-targeted radioimmunotherapy of ovarian cancer, namely, they prepared AR9.6-TCO and [^225^Ac]Ac-mcp-PEG_8_-Tz and evaluated pre-targeted method’s therapeutic efficacy and toxicity with normal radioimmunotherapy [149]. It was found that therapeutic efficacy of pre-targeted method using AR9.6-TCO and [^225^Ac]Ac-mcp-PEG_8_-Tz was as effective as normal method using [^225^Ac]Ac-mcp-PEG_8_-AR9.6 in OVCAR3 bearing mouse model, and pre-targeted method resulted in rapid blood and normal organ clearance of radioactivity leading to reduced toxicity demonstrating potential of pre-targeted radioimmunotherapy.

### Bi-213 and Pb-212

Bi-213 is a daughter radionuclide of Ac-225, with half-life of 45.6 min and α energy of 8.4 MeV. Because its short physical half-life does not match with long physiological half-life of full-length antibody, Bi-213 is not considered as an appropriate candidate radionuclide in the development of antibody-based radiopharmaceutical [150]. However, Bi-213-labeled full-length antibodies might be a better option to deliver radiation to widely disseminated tumors compared to less perfused subcutaneous tumor models in a preclinical study. Havlena et al. used disseminated tumor model by intravenous injection of CD20-expressing Raji cell, which expresses green fluorescence luciferase. They prepared Bi-213-labeled rituximab using CHX-A”-DTPA ([^213^Bi]Bi-Rituximab) and evaluated its therapeutic efficacy by measure fluorescent signal and survival of tumor mouse model [151]. It was found that two doses of [^213^Bi]Bi-Rituximab (75 μCi × 2) resulted in minimal fluorescence signal from the disseminated Raji cell indicating suppression of the tumor leading to cure of the cancer (50%, 3 out of 6 treated mice) facilitating potential of TAT against disseminated cancers.

Pb-212 is another alpha particle emitting radionuclide with half-life of 10.6 h, and it is considered in vivo generator due to serial alpha emissions from its daughter radionuclides (Bi212 and Po-212). Radioimmunotherapeutic agent utilizing Pb-212, [^212^Pb]Pb-TCMC-trastuzumab, for the treatment of HER2-expressing cancers has been clinically evaluated and it has shown promising result [18, 152]. Considering prostate cancer, radiolabeled PSMA-targeting peptides and antibodies, e.g. [^177^Lu]Lu-PSMA-617, [^177^Lu]Lu-J591, [^225^Ac]Ac-J591, have shown promising clinical efficacy. However, side effects such as xerostomia and heterogeneity of PSMA expression in mCRPC make TRT difficult and necessitate development of radiopharmaceuticals targeting more proper biomarkers [136, 153]. Li et al. discovered cell surface antigen, CD46, which is homogeneously expressed in prostate cancer, developed antibody against CD46, YS5, and evaluated in vivo therapeutic efficacy of Pb-212-labeled YS5 [154]. It was found that treatment of [^212^Pb]Pb-TCMC-YS5 (0.74 MBq) in PC3 subcutaneous mouse model resulted in significant inhibition of tumor growth leading to prolonged survival of experimental group (80% of the mice survived up to 55 days) compared to untreated control group (100% of the test mouse died within 37 days). They also evaluated therapeutic efficacy in PC3 orthotopic model as well as in PDX model, and the administration of [^212^Pb]Pb-TCMC-YS5 (0.74 MBq) significantly extended the survival of experimental group compared to the untreated control (100.5 days vs. 23 days and 99 days vs. 34 days in PC3 orthotopic model and PDX model, respectively) showing great potential of Pb-labeled radiopharmaceutical.

Another example of utilizing Pb-212 in a radiopharmaceutical is its use in pre-targeted radioimmunotherapy, which is more feasible due to relatively short half-life of Pb-212 (10.6 h). Pancreatic ductal adenocarcinoma (PDAC) is a lethal cancer with high mortality and limited treatment options, where 5-year survival rate is less than 10%, and the primary cause of death is metastatic relapse since PDAC is often at advanced stage when it is diagnosed [155]. TAT is considered appropriate option for the treatment of disseminated metastatic disease because it can deliver lethal dose of radiation to the cancer with minimal normal tissue toxicity. Carbohydrate cell surface antigen 19–9 (CA 19–9) is overexpressed in PDAC, and CA 19–9 targeting antibody 5B1 has been exploited by Bauer et al. for pre-targeted radioimmunotherapy using 5B1-TCO and [^212^Pb]Pb-DO3A-PEG_7_-Tz [156]. They showed administration of 5B1-TCO and following injection of [^212^Pb]Pb-DO3A-PEG_7_-Tz significantly inhibited tumor growth in BxPC-3 xenograft model and extended median survival to 8.3, 9.7, and 10.7 weeks, when 1.1, 2.2 3.7 MBq of radioligand was used, respectively, while median survival was 5.1 and 6.5 weeks for control groups of sole treatment of 5B1-TCO and administration of IgG-TCO followed by [^212^Pb]Pb-DO3A-PEG_7_-Tz (1.1 MBq), respectively, demonstrating therapeutic efficacy of pre-targeted RIT using Pb-212.

## Conclusion

Cancer diagnosis and therapy using radionuclides has been integral part of managing cancer since various radionuclides were produced vividly and cancer-specific biomarkers started to be discovered. Among different types of radiopharmaceuticals, antibody-based radiopharmaceuticals are the most studied one attributed to antibody’s exceptional target binding affinity. Antibody fragments including affibody and nanobody with improved pharmacokinetics compared to full-length antibody also have been studied frequently for the development of radiopharmaceuticals. In this review, the state-of-the-art studies in the development of diagnostic and therapeutic radiopharmaceuticals based on antibody and antibody fragments were introduced. Despite the fact that tremendous amount of research for developing radiolabeled nano-platforms has been conducted, there exist huge obstacles for them to overcome to be translated into clinics, and it is fair to say antibody and antibody fragments will continuously be the mainstream cancer targeting molecules in the development of radiopharmaceuticals.

## Data Availability

Not applicable.
